# Extension of a conditional performance score for sample size recalculation rules to the setting of binary endpoints

**DOI:** 10.1186/s12874-024-02150-4

**Published:** 2024-01-19

**Authors:** Björn Bokelmann, Geraldine Rauch, Jan Meis, Meinhard Kieser, Carolin Herrmann

**Affiliations:** 1 Charité - Universitätsmedizin Berlin, corporate member of Freie Universität Berlin and Humboldt-Universität zu Berlin, Institute of Biometry and Clinical Epidemiology, Charitéplatz 1, Berlin, 10117 Germany; 2 Institute of Medical Biometry, University Medical Center Ruprechts-Karls University Heidelberg, Im Neuenheimer Feld 130.3, 69120 Heidelberg, Germany; 3 Technische Universität Berlin, Straße des 17. Juni 135, 10623 Berlin, Germany

**Keywords:** Adaptive designs, Sample size recalculation, Performance score, Binary endpoint

## Abstract

**Background:**

Sample size calculation is a central aspect in planning of clinical trials. The sample size is calculated based on parameter assumptions, like the treatment effect and the endpoint’s variance. A fundamental problem of this approach is that the true distribution parameters are not known before the trial. Hence, sample size calculation always contains a certain degree of uncertainty, leading to the risk of underpowering or oversizing a trial. One way to cope with this uncertainty are adaptive designs. Adaptive designs allow to adjust the sample size during an interim analysis. There is a large number of such recalculation rules to choose from. To guide the choice of a suitable adaptive design with sample size recalculation, previous literature suggests a conditional performance score for studies with a normally distributed endpoint. However, binary endpoints are also frequently applied in clinical trials and the application of the conditional performance score to binary endpoints is not yet investigated.

**Methods:**

We extend the theory of the conditional performance score to binary endpoints by suggesting a related one-dimensional score parametrization. We moreover perform a simulation study to evaluate the operational characteristics and to illustrate application.

**Results:**

We find that the score definition can be extended without modification to the case of binary endpoints. We represent the score results by a single distribution parameter, and therefore derive a single effect measure, which contains the difference in proportions $p_{I}-p_{C}$ between the intervention and the control group, as well as the endpoint proportion $p_{C}$ in the control group.

**Conclusions:**

This research extends the theory of the conditional performance score to binary endpoints and demonstrates its application in practice.

**Supplementary Information:**

The online version contains supplementary material available at 10.1186/s12874-024-02150-4.

## Introduction

Sample size calculation is one central design aspect of clinical trials. The sample size is supposed to be large enough to detect existing effects but at the same time, there are multiple reasons why one should not choose an unnecessarily large sample size: The more patients are recruited, the higher the costs of the trial, the more patients are exposed to study-related risks and the longer the trial tends to be, which delays the launch of a potentially helpful medication on the market. The sample size needs to be determined before the start of a trial. It is calculated based on different parameter assumptions. Some of those parameters, e.g. the effect size, need to be determined based on previous study results and background knowledge. However, knowledge on those assumptions is often limited. One way of dealing with those difficulties regarding sample size calculation is to use adaptive group sequential study designs with the option to update the sample size during an ongoing trial. More explicitly, we will consider two-stage adaptive designs with one interim analysis. At this interim analysis, the effect size is estimated and either the trial can be stopped early for futility or efficacy, or the trial can be continued with potentially altering the sample size. There exists a great variety of sample size recalculation approaches in the literature [1–7]. Choosing an appropriate sample size recalculation approach out of the large number of options is a difficult task. Although the sample size recalculation approaches are usually published along with some performance assessments, the performance measures and the investigated scenarios vary across the literature. Most often, investigations are restricted to power and average sample size considerations. However, there exist also other important performance aspects to judge a recalculation approach, such as the variability of the recalculated sample size or the conditional power, which describes the probability of correctly rejecting the null hypothesis at the end of a trial when knowing the interim result. Moreover, despite pointing out advantages of adaptive designs and a whole paragraph on sample size adaptations, no explicit advice is given on the choice of sample size recalculation rules in the recent FDA guideline [8]. Similarly, the EMA reflection paper [9] provides no advice on how to select a certain sample size recalculation rule. To apply the recalculation rules in practice and to outweigh advantages and disadvantages in different approaches, the need for a fair and transparent performance comparison is of high importance. While Liu et al. [10] were one of the first suggesting a performance score for evaluating different sample size recalculation rules in adaptive study designs with respect to total sample size and overall power, Herrmann et al. [11] were the first who considered the conditional evaluation perspective and also scored the variation of conditional power and of recalculated sample size. The conditional evaluation perspective refers to those interim results that suggest a recalculation of the sample size and seems therefore very natural when comparing different sample size recalculation rules.

Herrmann et al. [11] defined and applied the conditional performance score to sample size recalculation approaches for normally distributed endpoints. In this paper, we extend their research to the situation of binary endpoints (e.g. [12, 13]). Thereby, we analyze how the score definition and the application procedure need to be adapted. Furthermore, we apply the same recalculation rules considered in [11] to the case of binary endpoints with two groups and assess their performance using the conditional performance score.

We find that the score definition itself can be extended without modification to the case of binary endpoints. That is, we can score the expected value and variation of conditional power and sample size in the same way as for normally distributed endpoints. However, unlike for normally distributed endpoints, common test statistics for binary endpoints (e.g., chi-square test, normal approximation test) have only asymptotically known distributions. Accordingly, specifying adaptive designs with efficacy stopping can lead to effects on the type I error rate. As for such designs the type I error rate depends on sample size and event rates [14], we need to analyze how these factors influence the type I error rate of the analyzed adaptive designs, to find possible limitations of their application.

Note that for normally distributed endpoints, the distribution of the test statistic only depends on a single parameter, the standardized treatment effect $\Delta$, while for binary endpoints, the distribution depends on the proportion in the control group $p_{C}$ and the treatment effect $p_{I}-p_{C}$, where $p_{I}$ is the proportion in the intervention group. Hence, we derive an alternative parameterization of the test statistic by a single parameter, which combines $p_{C}$ and $p_{I}-p_{C}$ into a single measure of effect size, fully specifying the distribution of the test statistic. Thereby, we allow for a simplified one-dimensional presentation of the score results and we enable direct comparison between the results of binary and normally distributed endpoints.

The paper is structured as follows: first, we introduce the considered adaptive designs, including the applied test statistic and the recalculation approaches. Then, we recall the conditional performance score and show how it can be extended to clinical trials with binary endpoints. Finally, we perform a simulation study, where we apply the conditional performance score as well as common global performance measures to the recalculation rules considered. We end by analyzing the results and giving advises for the practical application of the score.

## Adaptive designs and sample size recalculation

### Test problem

Throughout this work, we consider the situation of a randomized, controlled trial comparing an intervention group (I) with a control group (C). We focus on binary endpoints, where the respective endpoint distributions are given by Bernoulli distributions

$$\begin{aligned} \begin{array}{lll} X^{I}_{i}&{}\overset{iid}{\sim }\ {} &{} B(p_{I}),\\ X^{C}_{i}&{}\overset{iid}{\sim }\ {} &{} B(p_{C}). \end{array} \end{aligned}$$

Here, $p_{I}$ and $p_{C}$ denote the event probabilities for the binary endpoint in the intervention group *I* and the control group *C* respectively. In this paper, we consider the case of equal sample sizes *n* per group. We denote observations of both groups by $X^{I}_{i}$ and $X^{C}_{i}$, $i=1,...,n$ respectively. Without loss of generality, we assume that large proportions of the primary endpoint are favourable. The test problem to be assessed in confirmatory analysis is thus given by

1 $$\begin{aligned} H_{0}:p_{I}-p_{C}\le 0\ \text {versus}\ H_{1}:p_{I}-p_{C}>0. \end{aligned}$$

To test the null hypothesis, we apply the normal approximation test. The test statistic is defined by

2 $$\begin{aligned} Z=\sqrt{\frac{n}{2}}\frac{\bar{X}^{I}-\bar{X}^{C}}{\sqrt{\frac{\bar{X}^{I}+\bar{X}^{C}}{2}(1-\frac{\bar{X}^{I}+\bar{X}^{C}}{2})}}, \end{aligned}$$

where $\bar{X}^{I}, \bar{X}^{C}$ denote the observed proportions. Note that the following results can be extended to the use of the chi-square test, since the test statistic of the normal approximation test is just the square root of the chi-square test statistic.

We assume a sufficiently large sample size, such that the distribution of *Z* is given by

3 $$\begin{aligned} Z\sim N\left( \frac{p_{I}-p_{C}}{\sqrt{(\frac{p_{I}+p_{C}}{2})(1-\frac{p_{I}+p_{C}}{2})}}\sqrt{\frac{n}{2}},\frac{p_{I}(1-p_{I})+p_{C}(1-p_{C})}{2(\frac{p_{I}+p_{C}}{2})(1-\frac{p_{I}+p_{C}}{2})}\right) , \end{aligned}$$

cf. Appendix A for details.

### Parameterization of the test statistic distribution

The distribution of the test statistic given in Eq. (3) depends, apart from the per-group sample size *n*, on two distribution parameters $p_{I}$ and $p_{C}$. Accordingly, we call the representation given in (3) a two-dimensional parameterization of the distribution of *Z*. In fact, it is also possible to parameterize the distribution of *Z* by a single parameter. Appendix B shows the derivation of a one-dimensional parameterization of the distribution of *Z*, which is then given by

4 $$\begin{aligned} Z\sim N\left( \lambda \sqrt{\frac{n}{2}},1-\frac{1}{4}\lambda ^2\right) , \end{aligned}$$

where $\lambda$ is defined as

5 $$\begin{aligned} \lambda {:=}\frac{p_{I}-p_{C}}{\sqrt{\frac{p_{I}+p_{C}}{2}\left( 1-\frac{p_{I}+p_{C}}{2}\right) }}. \end{aligned}$$

**Fig. 1:**
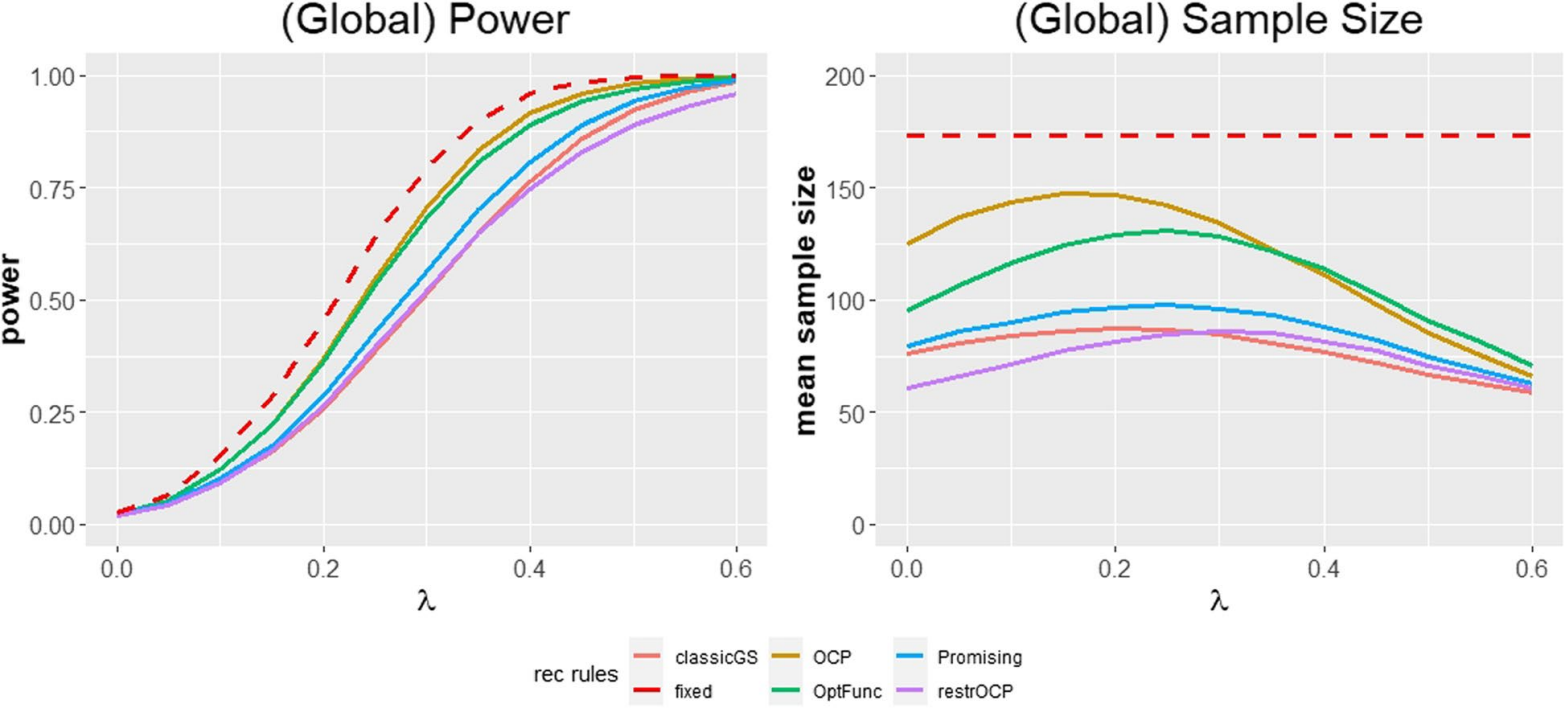
Global power and mean sample size for the considered recalculation rules for binary endpoints in the main simulation. The red dashed line represents the sample size and power for a fixed design with a target power of 80% for an assumed $\lambda =0.3$. The proportion in the control group is set to $p_{C}=0.3$ and the first stage sample size is set to $n_{1}=50$. The global significance level $\alpha = 0.025$, efficacy stopping boundaries were chosen according to Pocock [15] and the binding futility stopping bound is set to $\alpha _{0}=0.5$. The maximum sample size is set to $n_{max} = 200$. OCP: Observed conditional power approach; restrOCP: Restricted observed conditional power approach; Promising: Promising zone approach; OptFunc: Optimization function approach; classicGS: group sequential approach

**Fig. 2:**
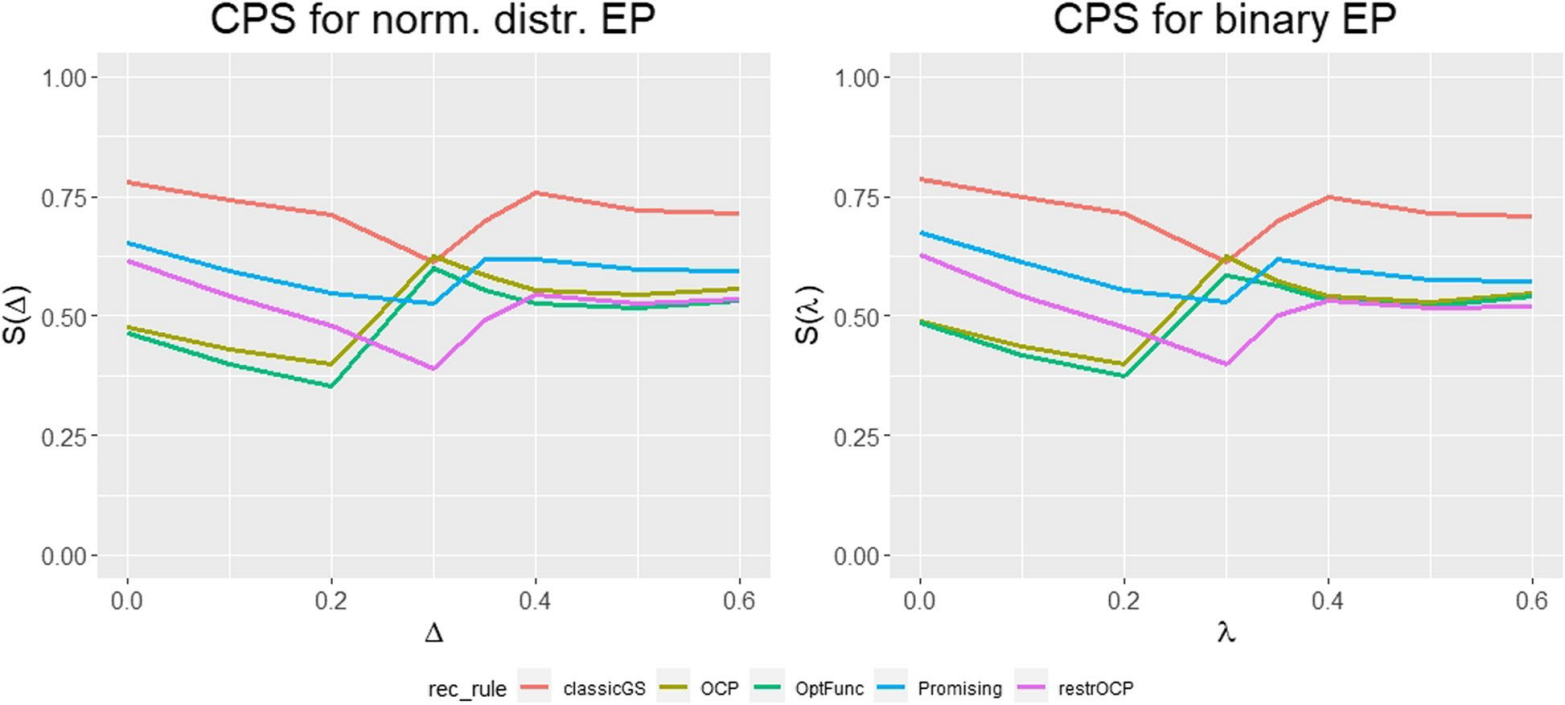
Comparison of the conditional performance score (CPS) values between normally distributed endpoints (left panel) and binary endpoints (right panel) for the considered sample size recalculation rules in the main simulation. Score values are printed for $\Delta ,\lambda \in \{0,0.1,0.2,0.3,0.35,0.4,0.5,0.6\}$. The first stage sample size is set to $n_{1}=50$, the maximum sample size is set to $n_{max}=200$ with global one-sided significance level $\alpha = 0.025$, efficacy stopping boundaries were chosen according to Pocock [15] and binding futility stopping bound represented by $\alpha _{0}=0.5$. OCP: Observed conditional power approach; restrOCP: Restricted observed conditional power approach; Promising: Promising zone approach; OptFunc: Optimization function approach; classicGS: group sequential approach

The one-dimensional parameterization of the distribution of *Z* is more concise than the two-dimensional parameterization. The parameter $\lambda$ can be interpreted as the standardized treatment effect for binary endpoints. In the remainder of the paper, we provide various formulas (for the conditional power of adaptive designs, for the required fixed design sample size, ...) which all contain the parameter $\lambda$. This is because these formulas are derived from the test statistic distribution. These formulas only contain $\lambda$ and neither $p_{I}$ nor $p_{C}$. Accordingly, the formulas yield for a given $\lambda$ always the same results, irrespective of the concrete values of $p_{I}$ and $p_{C}$. This property has two favorable consequences: First, we can present performance results (like power, mean sample size and conditional performance score) as a function of a single effect size parameter $\lambda$. This enables us to provide the results for a performance measure in a single plot (see Figs. 1 and 2). If we instead chose to present performance results depending on the effect size $p_{I}-p_{C}$, the results would differ, depending on the underlying value $p_{C}$. Hence, we would need to generate separate plots for each plausible value of $p_{C}$. Second, the parameterization by $\lambda$ enables us to relate our results for binary endpoints to the results for normally distributed endpoints by Herrmann et al. [11]. This is because the authors present their results depending on the standardized treatment effect $\Delta$ for normally distributed endpoints, which plays a similar role as the parameter $\lambda$ in this paper. However, the parameterization by $\lambda$ has the disadvantage of losing some interpretability. Clinicians know the meaning of a control group endpoint proportion of $p_{C}=0.3$ and a treatment effect of $p_{I}-p_{C}=0.12$ but might have problems interpreting $\lambda =0.25$, which is equivalent. To show which combinations of the control group proportion $p_{C}$ and the treatment effect $p_{I}-p_{C}$ are represented by a certain $\lambda$, we provide Table 1.

**Table 1:** Treatment effects $p_{I}-p_{C}$, corresponding to combinations of $p_{C}$ and $\lambda$. The table shows which combinations of $p_{C}$ and $p_{I}-p_{C}$ correspond to a given value of $\lambda$. A cell at row *i* and column *j* corresponds to the value $p_{I}-p_{C}$ for the *i-th* value of $\lambda$ and the *j-th* value of $p_{C}$

	$$p_{C}$$							
	0	0.02	0.05	0.1	0.2	0.3	0.4	0.5
$$\varvec{\lambda }$$								
0	0	0	0	0	0	0	0	0
0.05	0.001	0.008	0.012	0.016	0.020	0.023	0.025	0.025
0.1	0.005	0.017	0.024	0.032	0.042	0.047	0.049	0.050
0.15	0.011	0.027	0.038	0.050	0.063	0.071	0.074	0.075
0.2	0.020	0.039	0.053	0.068	0.086	0.095	0.100	0.100
0.25	0.031	0.053	0.070	0.088	0.109	0.120	0.125	0.124
0.3	0.044	0.068	0.087	0.108	0.133	0.145	0.150	0.148
0.35	0.059	0.085	0.107	0.130	0.157	0.170	0.175	0.172
0.4	0.077	0.103	0.127	0.152	0.182	0.196	0.200	0.196
0.45	0.096	0.123	0.148	0.176	0.207	0.221	0.225	0.220
0.5	0.118	0.145	0.171	0.200	0.233	0.247	0.250	0.243
0.55	0.141	0.168	0.195	0.225	0.259	0.273	0.274	0.265
0.6	0.165	0.192	0.220	0.251	0.285	0.298	0.299	0.287

The one-dimensional parameterization by $\lambda$ is derived from the asymptotic distribution of *Z* (see Test problem section and Appendix B). For the distribution of the test statistic *Z* and the derived parameterization, we assumed a large enough sample size to assume normality. Since in practice, the sample size is finite and the assumed distribution of our test statistic *Z* holds only approximately, it is necessary to analyse whether the theory of the one-dimensional parameterization is applicable. This is done in Appendix E, where we show that our theory of the one-dimensional parameterization holds approximately even for rather small sample sizes.

### Two-stage adaptive designs

For analyzing the one-dimensional parametrization by $\lambda$ in practice, we consider the following setting:

1. The adaptive design is planned for two stages with one interim analysis. We allow to stop for efficacy or futility at interim. If the trial is not stopped, recalculation of sample size for the second stage is performed.
2. We assume equal sample sizes in the intervention and control group during both stages. We denote the per-group sample size at the interim analysis by $n_{1}$ (yielding a total sample size of $2\cdot n_{1}$ at stage one) and the per-group second stage sample size by $N_{rec}$ (yielding a total sample size of $2\cdot N_{rec}$ at stage two). The according per-group total sample size *N* is then given by $n_1 + N_{rec}$.
3. We apply the same kind of test statistic at both stages. Accordingly, we denote the test statistic at interim by $Z_{1}$ and at the second stage by $Z_{2}$. Both test statistics can be derived from the general test statistic *Z*, by specifying the per-group sample size $n_{1}$ for $Z_{1}$ and $N_{rec}$ for $Z_{2}$.
4. Decisions about stopping for futility or efficacy at the first stage are based on $Z_{1}$. We only consider binding futility stops. Decisions about the acceptance or rejection of the null hypothesis at the second stage are based on a combination $Z_{1+2}$ of both $Z_{1}$ and $Z_{2}$. The form of $Z_{1+2}$ will be derived below.

For our combined test statistic $Z_{1+2}$, we use the inverse normal combination test (cf. e.g. [16]). Due to the standard normal distribution of our test statistic *Z* under the null hypothesis, the inverse normal combination test has the simplified form

$$\begin{aligned} Z_{1+2}{:=}\frac{w_{1}Z_{1}+w_{2}Z_{2}}{\sqrt{w_{1}^{2}+w_{2}^{2}}}. \end{aligned}$$

Here, $w_{1},w_{2}$ are weights, which are fixed before starting the trial. Under the null hypothesis, $Z_{1+2}$ follows the standard normal distribution.

Since we allow for rejection of the null hypothesis at the first and second stage, we need to specify the local significance levels $\alpha _{1}$ and $\alpha _{1+2}$, such that the null hypothesis is rejected if $Z_{1}\ge q_{1-\alpha _{1}}$ or $Z_{1+2}\ge q_{1-\alpha _{1+2}}$ and at the same time the global type I error rate $\alpha$ is not exceeded. Here, *q* denotes the quantiles of the standard normal distribution. Given, that the covariance is asymptotically given by $cov(Z_{1},Z_{1+2})=\frac{w_{1}}{\sqrt{w_{1}^{2}+w_{2}^{2}}}$ and knowing that $Z_{1}$ and $Z_{1+2}$ are both asymptotically standard normally distributed under the null hypothesis, we know the asymptotic distribution of $(Z_{1},Z_{1+2})$ and can thereby calculate local signficance levels which maintain the type I error rate. Here, we choose the Pocock boundaries $\alpha _{1}=\alpha _{1+2}$, such that the global one-sided significance level $\alpha =0.025$ is maintained (cf. e.g. [15]). We further choose $\alpha _{0}=0.5$, such that the trial is stopped for futility, if $Z_{1}<q_{1-\alpha _{0}}$.

### Sample size recalculation

Until now, we introduced $n_{1}$ and $N_{rec}$ as the per-group sample sizes of the first and second stage of the trial. The first stage per-group sample size $n_{1}$ is chosen before the beginning of the trial, while $N_{rec}$ is chosen after interim results are obtained. In this section, we present common principles for how to choose $N_{rec}$ based on the interim results. A plausible decision criterion is the conditional power, which will be described in the following. After introducing the concept of the conditional power, we describe the recalculation rules applied in the following simulation.

#### Conditional power

The conditional power is the probability to correctly reject the null hypothesis at the second stage, given the observed interim data (cf. e.g. [17]). This probability is zero, if the trial is stopped early for futility. If the trial continues with the second stage, then the conditional power (CP) is defined as

6 $$\begin{aligned} CP_{\lambda }(Z_{1},N_{rec}){:=}P_{\lambda }[Z_{1+2}\ge q_{1-\alpha _{1+2}}|Z_{1}]. \end{aligned}$$

This probability depends on the result of the interim test statistic $Z_{1}$ as well as on the recalculated sample size $N_{rec}$ (which affects the distribution of $Z_{1+2}$). Note the use of the distribution parameter $\lambda$ in this equation. Here, $\lambda$ specifies the distribution of *Z* and thereby the distribution of $Z_{1+2}$.

The conditional power is a plausible criterion to decide about the second stage sample size $N_{rec}$ (cf. e.g. [17]). The basic idea is to choose $N_{rec}$ large enough, such that a targeted conditional power is achieved. However, researchers do not know the value of the distribution parameter $\lambda$, which would be required to calculate the (true) conditional power. Following Wassmer and Brannath [18], there are multiple ways to estimate the parameter value for the conditional power, based on the interim results. Regarding our distribution parameter $\lambda =(p_{I}-p_{C})/(\sqrt{\frac{p_{I}+p_{C}}{2}\left( 1-\frac{p_{I}+p_{C}}{2}\right) })$, it would be possible to keep the numerator $p_{I}-p_{C}$ fixed as a minimally clinical relevant effect and only estimate the denominator, which corresponds to the endpoint’s standard deviation. Another approach would be to use a Bayesian estimation approach to combine prior knowledge with the interim results (cf e.g. [19]). A third approach is to use the observed standardized treatment effect $\hat{\lambda }$, which is given by

7 $$\begin{aligned} \hat{\lambda }{:=}\frac{\bar{X}^{I}-\bar{X}^{C}}{\sqrt{\frac{\bar{X}^{I}+\bar{X}^{C}}{2}(1-\frac{\bar{X}^{I}+\bar{X}^{C}}{2})}}. \end{aligned}$$

We restrict our analysis to recalculation rules relying on the observed standardized treatment effect $\hat{\lambda }$. Accordingly, each recalculation rule is based on the observed conditional power $CP_{\hat{\lambda }}(Z_{1},N_{rec})$.

#### Considered recalculation approaches

In the following, we present different recalculation approaches, which will be applied in the simulation study. All the approaches have in common, that we define a certain maximum per-group sample size $n_{max}$, such that the recalculated per-group sample size $N_{rec}$ needs to be lower or equal to $n_{max}-n_{1}$. Accordingly, the total sample size of the trial is lower than $2\cdot n_{max}$. We set $n_{max}$ to the same value for each recalculation approach. Recalculation is only performed if $Z_{1}$ lies in an interval, where we neither stop for futility or efficacy. This interval is called recalculation area.

Apart from a constant second stage sample size within the recalculation area (classic group sequential approach), we consider four other sample size recalculation approaches in the following simulation study:

- **Observed conditional power approach**: The underlying idea of the observed conditional power approach is to update the sample size such that a specified conditional power value $1-\beta$ is achieved. Whenever the recalculated sample size exceeds the maximal per-group sample size, $N_{rec}=n_{max}-n_{1}$ is chosen instead.
- **Restricted observed conditional power approach**: The restricted observed conditional power approach is to be seen as an extension of the observed conditional power approach. It could be considered as not being worth the effort to choose the maximal sample size if only a conditional power smaller than $1-\beta _{0}^{restrOCP} \le 1-\beta$ can be obtained. Whenever this is the case, the sample size is not increased and the trial stops after the first stage instead.
- **Promising zone approach**: The promising zone approach was proposed by Mehta and Pocock [5] and can be seen as a combination of the classical group sequential and the observed conditional power approach. In the so called “unfavourable zone”, when the conditional power for the sample size $n_{GS}$ of the classic group sequential study design falls below a certain conditional power threshold $1-\beta _{0}^{prom}$, the group sequential sample size $n_{GS}$ is chosen. In the “favourable zone”, when the conditional power for $n_{GS}$ is larger than $1-\beta$, again the constant sample size $n_{GS}$ is chosen. For all conditional power values in between $1-\beta _{0}^{prom}$ and $1-\beta$, the called “promising zone”, the observed conditional power approach is applied.
- **Optimization function approach**: Jennison and Turnbull [6] suggested optimizing a function to recalculate the sample size. More precisely, they defined a function *f* in dependence of the recalculated sample size, where its scaled deviation from the classic group sequential sample size is set off against the gain in conditional power. The recalculated sample size that optimizes that function *f* is then chosen as recalculated sample size. This sample size is again bounded from above by $n_{max}$.

Detailed descriptions of the recalculation rules can be found in [11]. The corresponding formulas are given in Appendix C.2.

## Evaluation of adaptive designs with sample size recalculation

To judge and compare sample size recalculation approaches, different performance measures can be used. Commonly, the expected sample size and power are used. These, however, should always be considered together with measures of variation since a design that performs *on average* well, cannot be judged as good if it is always missing the target values by far [11]. Hence, measures of location and variation of the sample size and power are to be included. The definition of target values for the location and variation measures is in general not straightforward. Liu et al. [10] and Herrmann et al. [11] give suggestions for their scores. The two performance scores differ in that Liu et al. [10] do not consider variation measures in their score definition and they differ with respect to their evaluation perspective: Liu et al. [10] consider the *global* perspective, which refers to any interim result that either suggests a continuation or stopping of the trial. Herrmann et al. [11] argue that it is natural to also consider the so called *conditional* perspective, which refers to all results where the interim test statistic falls in the recalculation area when judging the performance of a sample size recalculation approach. Both perspectives are valid and required simultaneously. In the following, we focus on the extension of the conditional performance score to binary endpoints and therefore present this score in more detail.

### Definition of the conditional performance score

The conditional performance score *S* was suggested by Herrmann et al. [11] and can be used for the comparison of different sample size recalculation approaches under the assumption of observing an interim effect falling in the recalculation area, i.e., $z_1 \in [q_{1-\alpha _0}; q_{1-\alpha _1}]$. Its core underlying idea is to evaluate the conditional power and total recalculated per-group sample size (given by $N = n_1 + N_{rec}$) with respect to location (*l*) and variation (*v*). These four components are then combined by

8 $$\begin{aligned} S (\lambda ) = \frac{1}{4} \cdot l_{CP}(\lambda ) + \frac{1}{4} \cdot v_{CP}(\lambda ) + \frac{1}{4} \cdot l_{N}(\lambda ) + \frac{1}{4} \cdot v_{N}(\lambda ) , \end{aligned}$$

where $\lambda$ refers again to the parameterization. Note, that it is also possible to choose other weights than the constant 1/4 for the score components, if one wants to make any of the four components more important than the others in the evaluation. In this paper, however, we use the constant weights of 1/4 throughout. The score itself as well as all of its four components can take values between 0 and 1, where higher values correspond to a better performance. Hence, not only the total score value can be interpreted but also a differentiated argumentation regarding the four evaluation criteria can be provided. More precisely, the idea of the location components is to evaluate the difference of the expected value (conditional power or recalculated sample size) from a corresponding target value divided by the maximal possible difference. For the conditional power, it is given by

$$\begin{aligned} l_{CP}(\lambda ) = 1-\frac{|E[CP(Z_1, N_{rec})]-CP_{target, \lambda }|}{1-\alpha }. \end{aligned}$$

Here, $CP_{target, \lambda }$ is the target value for the conditional power. It takes different values (namely the aspired conditional power $1-\beta$ or the significance level $\alpha$) depending on whether the effect $\lambda$ is large enough to conduct a trial with sufficient power while maintaining the maximum sample size $n_{max}$. Equivalently,

$$\begin{aligned} l_{N}(\lambda ) = 1-\frac{|E[N]-N_{target, \lambda }|}{n_{max}-n_{1}} \end{aligned}$$

describes the location component for the recalculated sample size. Here, $N_{target,\lambda }$ is the target value for the sample size. It also takes different vales (namely the fixed design per-group sample size $n_{fix}(\lambda )$ for a power of $1-\beta$ or the first stage per-group sample size $n_{1}$), again depending on whether the effect $\lambda$ is large enough to conduct a trial with sufficient power while maintaining the maximum sample size $n_{max}$. Details of the theory behind target values are provided in [11] and explicit values for them are given in Table 2.

**Table 2:** Target values, maximal possible deviations from the target values, and variances for the conditional performance score. CP: conditional power; *N*: total recalculated sample size; $1-\beta$: anticipated conditional power value; $\alpha$: significance level; $n_{fix}(\lambda )$: sample size for $\lambda$ in fixed sample size design; $n_1$: first stage sample size; $n_{max}$: maximal sample size

Performance measure	target value for $\varvec{n}_{\varvec{fix}}(\varvec{\lambda }) \varvec{\le } \varvec{n}_{\varvec{max}}$ and $\varvec{\lambda >0}$ (target value for $\varvec{n}_{\varvec{fix}}(\varvec{\lambda }) \varvec{>} \varvec{n}_{\varvec{max}}$) and $\varvec{\lambda \le 0}$	maximal possible deviation from target value	maximal possible variance
*CP*	$1-\beta$ ($\alpha$)	$1-\alpha$	${\left( (1-0)/2 \right) }^2$
*N*	$n_{fix}(\lambda ) (n_1)$	$n_{max}-n_1$	${\left( (n_{max}-n_1)/2 \right) }^2$

The variation components evaluate the ratio of the observed variances and the maximal possible variance with

$$\begin{aligned} v_{CP}(\lambda ) = 1- \sqrt{\frac{Var(CP(Z_1, N_{rec}))}{1/4}}, \end{aligned}$$

and

$$\begin{aligned} v_{N}(\lambda ) = 1- \sqrt{\frac{Var(N)}{{\left( (n_{max}-n_1)/2 \right) }^2}}. \end{aligned}$$

We refer to [11] for the derivation of the maximal possible variances 1/4 and ${\left( (n_{max}-n_1)/2 \right) }^2$.

As for most kinds of performance scores, a clear interpretation of the resulting score values is difficult to provide. However, a coarse guideline is given in [11].

Note that the definition of the conditional performance score does not impose any requirements with regard to the distribution of the endpoints. This means that the expected value and variance of *N* and *CP* exist for normally distributed endpoints, for binary endpoints, time-to-event endpoints and so on. Accordingly, the definition itself of the conditional performance score, given for normally distributed endpoints by Herrmann et al. [11], does not require any adaption to be applied on binary endpoints. What needs to be adapted to the other endpoint type is how to calculate the score.

### Calculation of the conditional performance score for binary endpoints

When a scientist wants to apply the conditional performance score to evaluate a recalculation rule, s/he needs to know how to apply Eq. (8). For the target values $CP_{target,\lambda }$ and $N_{target,\lambda }$, explicit values are provided in Table 2. However, to calculate the score, the scientist still needs to know how to calculate the expected value and the variance of the sample size *N* and the conditional power *CP*, as well as the fixed design sample size $n_{fix}$.

To calculate the fixed design sample size $n_{fix}$, a formula depending on the endpoint distribution parameters is required. Appendix D shows how the formula

9 $$\begin{aligned} n_{fix}(\lambda )=\left( \sqrt{2}q_{1-\alpha _{1+2}}\frac{1}{\lambda }+q_{1-\beta }\sqrt{\frac{2}{\lambda ^{2}}-\frac{1}{2}}\right) ^{2} \end{aligned}$$

can be derived for binary endpoints with the parameterization based on $\lambda$.

To calculate the expected value and variance of the sample size *N*, the scientist needs to simulate the distribution of *N*. Since $N=n_{1}+N_{rec}$ and only $N_{rec}$ is stochastic, the scientist only needs to simulate the distribution of $N_{rec}$. To simulate the distribution of $N_{rec}$, s/he needs to simulate the distribution of the interim test statistic and apply the formula of a considered recalculation rule to calculate $N_{rec}$ from the interim results. For all the recalculation rules considered in this study, formulas $N_{rec}=N_{rec}(Z_{1})$ for the respective approaches, which only depend on the interim results $Z_{1}$, are provided in Appendix C.2. Given these formulas and the simulated distribution of $Z_{1}$, the scientist can simulate the distribution of *N* and thereby calculate its expected value and variance.

To calculate the expected value and variance of the observed conditional power, the scientist needs to simulate the distribution of the observed conditional power. To this end, s/he needs a formula for the observed conditional power

10 $$\begin{aligned} CP_{\hat{\lambda }}(Z_{1},N_{rec})=1-\Phi \left( \frac{\frac{\sqrt{w_{1}^{2}+w_{2}^{2}}}{w_{2}}q_{1-\alpha _{1+2}}-\frac{w_{1}}{w_{2}}Z_{1}-\hat{\lambda }\sqrt{\frac{N_{rec}}{2}}}{\sqrt{1-\frac{1}{4}\hat{\lambda }^{2}}}\right) , \end{aligned}$$

with $\hat{\lambda }$ respresenting the observed standardized effect size and $\Phi$ being the cumulative distribution function of the standard normal distribution. More details can be found in Appendix C.1. Given a simulated distribution of $Z_{1}$, combined with the corresponding distribution of $N_{rec}$, described in the above paragraph, the scientist can generate a simulated distribution of $CP_{\hat{\lambda }}(Z_{1},N_{rec})$ and then calculate its expected value and variance.

Note that for calculating $n_{fix}(\lambda )$ we only need $\lambda$ and for the distributions of $N_{rec}$ and $CP_{\hat{\lambda }}(Z_{1},N_{rec})$ we only need the distribution of $Z_{1}$. Hence, we only need $\lambda$ and the distribution of $Z_{1}$ for calculating the conditional performance score.

## Simulation study

Our simulation study contains two parts. In the first part, the simulation setting is similar to the main simulation setting in the study by Herrmann et al. [11], except that we apply binary endpoints instead of normally distributed endpoints. This simulation is meant to show the relationship between score results for both endpoint types. We refer to this first simulation as the “main simulation”. The second part simulates the application of recalculation rules and the conditional performance score to a real-world trial with binary endpoints, namely the APSAC trial [20]. This trial had initially a fixed sample size design. Our simulation shows, how the trial would have been different if, instead of the fixed design, adaptive designs with sample size recalculation would have been applied. Thereby, we demonstrate the effect of sample size recalculation and scoring for a realistic trial example. We refer to this simulation as the “APSAC simulation”.

### Simulation settings

First, we specify all parameter values underlying the main simulation for the comparison of performance score results for binary and normally distributed endpoints. We score adaptive designs relying on the observed conditional power approach, the restricted observed conditional power approach, the promising zone approach, the optimization function approach, and the classic group sequential approach respectively. We set the initial per-group sample size to $n_{1}=50$ and the maximum per-group sample size to $n_{max}=200$. The global one-sided significance level $\alpha$ is set to 0.025 with adjustment for multiple testing according to Pocock [15] (i.e. $\alpha _{1}=\alpha _{1+2}=0.0147$) and futility stopping boundary $\alpha _0 = 0.5$. Our targeted conditional power is $1-\beta =0.8$. Weights of the inverse normal combination test are given by $w_{1}=w_{2}=\sqrt{n_{1}} = \sqrt{50}$. In this way, the weight of the two stages in the inverse normal combination test corresponds to the sample sizes of the two stages for the group sequential design. This choice of weights would then lead to the highest power the group sequential design could achieve with $n_{1}=n_{2}=50$. Specific parameters for the sample size recalculation approaches (cf. Considered recalculation approaches section and Appendix C.2) are given by $\beta _{0}^{restrOCP}=0.4$ for the restricted observed conditional power approach, $\beta _{0}^{Prom}= 0.64$, $n_{ini}^{Prom}=n_{1}=50$ for the promising zone approach. For the optimization function approach, we apply the trade-off value $\gamma =0.005/4$ between conditional power and sample size (see [6] for details). We further set the initially planned per-group second stage sample size $n_{ini}^{OptFunc}=n_{1}=50$. As a software, we use R [21] (version 3.6.1).

Now, we describe the APSAC simulation. The APSAC trial, which we choose as a real-world trial example, aimed to measure the effect of APSAC medication compared to Heparin medication for patients with acute myocardial infarction [20]. Primary endpoint of the study was hospital mortality within 28 days. For sample size calculation, a 4% mortality for the APSAC patients and a 12% mortality for the Heparin patients were assumed, which results in 180 patients per treatment arm for a power of $80\%$ and a one-sided significance level of $2.5\%$. While the actual trial had a fixed sample size design, a two-stage adaptive design can especially be of interest when there is a high pre-trial insecurity about the mortality in the two groups. Therefore, we simulated two-stage adaptive designs with a first stage per-group sample size of $n_{1}=90$. We set the maximum per-group sample size to $n_{max}=270$. The assumed mortality rates of 4% and 12% correspond to a standardized treatment effect of $\lambda = -0.295$. Note that, in the definition of the test problem, we assumed without loss of generality, that the treatment would aim for an increase in the proportion of the primary endpoint. To simulate the APSAC trial, where the treatment has a negative effect on the endpoint proportion, we needed to switch the role of $p_{I}$ and $p_{C}$ in the according equations and in the simulation parameters. Accordingly, the assumed standardized treatment effect applied in the simulation was $\lambda = 0.295$. The remaining simulation parameters were chosen as in the main simulation. Only for the optimization function approach, we chose $\gamma =0.0022$. The value was chosen as $\gamma = Pow_{\lambda }(n_{fix})-Pow_{\lambda }(n_{fix}-1)$, where $Pow_{\lambda }$ denotes the power of a fixed design for a given sample size. The idea behind this choice was to set the “cost” of each unit in sample size, such that the optimal sample size according to the optimization function approach would approximately correspond to the sample size needed to achieve a power of $80\%$.

### Calculation of performance measures

In the simulation study, we applied three different performance measures: the conditional performance score, the global average sample size, and the global power. The performance measures were calculated by simulation. Below, we provide the respective details.

To calculate the conditional performance score, we needed the simulated distribution of $Z_{1}$ (see Calculation of the conditional performance score for binary endpoints section). Therefore, we simulated $N_{sim}=10.000$ observations of $Z_{1}$ for each of the three first stage sample sizes $n_{1}=10, 20, 50, 90$. We simulated the exact distribution of $Z_{1}$, based on Bernoulli random numbers and Eq. (2). Having simulated the distribution of $Z_{1}$, we followed the steps in Calculation of the conditional performance score for binary endpoints section to calculate the conditional performance score.

The global average sample size was calculated as a by-product of the sample size component of the conditional performance score. Since the calculation of the conditional performance score involves the simulation of the total per-group sample size N for each simulation run (see Calculation of the conditional performance score for binary endpoints section).

In Appendix C.3, we show that the global power can be estimated, via simulation, according to

$$\begin{aligned} Pow(\lambda )=\frac{1}{N_{sim}}\sum _{i=1}^{N_{sim}}(\mathbbm {1}_{Z_{1,i}\ge q_{1-\alpha _{1}}}+\mathbbm {1}_{q_{1-\alpha _{0}}\le Z_{1,i} < q_{1-\alpha _{1}}}\cdot CP_{\lambda }(Z_{1,i},N_{rec,i})), \end{aligned}$$

where $Z_{1,i}$ denote the values of the interim test statistic of simulation $i\in \{1,2,...,N_{sim}\}$. This power calculation requires the true conditional power $CP_{\lambda }(Z_{1,i},N_{rec,i})$. To calculate the true conditional power we did not use an analytical approach (as such an approach would rely on the asymptotic distribution of the test statistic) but simulated the true conditional power. We used the formula

11 $$\begin{aligned} CP_{\lambda }(Z_{1},N_{rec})=P_{\lambda }\Biggl [Z_{2}>\frac{q_{1-\alpha _{1+2}}\sqrt{w_{1}^{2}+w_{2}^{2}}-w_{1}Z_{1}}{w_{2}}\Biggr ], \end{aligned}$$

which can easily be derived from Eq. (6). For given $Z_{1}$ and $N_{rec}$, we then calculated the conditional power by simulating $Z_{2}$ and obtained the share of cases where it surpasses $(q_{1-\alpha _{1+2}}\sqrt{w_{1}^{2}+w_{2}^{2}}-w_{1}Z_{1})/w_{2}$.

### Results main simulation

In the following, we present the results for the global sample size, the global power and the conditional performance score for values of the standardized treatment effect $\lambda$ ranging from 0 to 0.6. In the main setting below we chose a control group proportion of $p_{C}=0.3$. However, the results for other control group proportions are very similar, as can be seen in Appendix E. This similarity can be explained by our thoughts presented in Parameterization of the test statistic distribution section.

Before analyzing the conditional performance score values in detail, we examine the designs from the global perspective. Figure 1 shows the average sample size and global power of the five adaptive designs as well as the fixed design for an assumed standardized treatment effect of $\lambda =0.3$. We can see that the average sample size of all the adaptive designs remains constantly below the sample size of the fixed design (with power values likewise). For very low ($\lambda \le 0.1$) and very large ($\lambda \ge 0.5$) standardized treatment effects, the difference in power between most adaptive designs and the fixed design is rather small. This shows benefits of adaptive designs compared to the fixed design because sample size is used more efficiently for these standardized treatment effects.

A second insight from the global perspective is that for $\lambda \le 0.1$, all the adaptive designs have a low power with values below 13%. At the same time, average sample size values of these designs are still comparatively high in this range (although lower than for the fixed design). The combination of comparatively high sample size values and low power corresponds to a “waste of sample size” to a futile trial. This is important to know as it indicates that none of the designs should be used if the true standardized treatment effect is likely to lie in this range.

Now, we switch to the conditional perspective and examine the conditional performance score results. Figure 2 shows the simulation results for binary endpoints in comparison with the simulation results from Herrmann et al. [11] for normally distributed endpoints. Table 3 shows the exact values for binary endpoints underlying the figure. The score results for normally distributed and binary endpoints are very similar. There are minor shifts, especially for relatively large values of the standardized treatment effects ($\Delta , \lambda$). However, in general, observations for the case of normally distributed endpoints made by Herrmann et al. [11] can be extended to the case of binary endpoints: For example, the group sequential design performs best according to the conditional performance score, which can be attributed to the lower variation in the sample size, compared to the other recalculation rules. Furthermore, the ranking between the other designs remains stable for $\lambda$ between 0 and 0.2, then changes for $\lambda$ between 0.2 and 0.4 and then changes again. For a detailed analysis of the results, we refer to the paper by Herrmann et al. [11].

**Table 3:** Score results $S(\lambda )$ for binary endpoints in the main simulation. The values in the table correspond to Fig. 2 for binary endpoints. The first stage sample size is set to $n_{1}=50$, the maximum sample size is set to $n_{max}=200$ with global one-sided significance level $\alpha = 0.025$, efficacy stopping boundaries were chosen according to Pocock [15] and binding futility stopping bound represented by $\alpha _{0}=0.5$. OCP: Observed conditional power approach; restrOCP: Restricted observed conditional power approach; Promising: Promising zone approach; OptFunc: Optimization function approach; classicGS: group sequential approach

$\varvec{\lambda }$	OCP	restrOCP	Promising	OptFunc	classicGS
0	0.488	0.628	0.673	0.485	0.785
0.05	0.463	0.586	0.639	0.452	0.767
0.1	0.437	0.540	0.612	0.418	0.748
0.15	0.417	0.503	0.580	0.394	0.732
0.2	0.400	0.476	0.553	0.373	0.715
0.25	0.387	0.451	0.526	0.356	0.698
0.3	0.624	0.400	0.527	0.585	0.612
0.35	0.573	0.502	0.617	0.562	0.700
0.4	0.542	0.531	0.601	0.532	0.750
0.45	0.532	0.516	0.582	0.525	0.728
0.5	0.530	0.516	0.574	0.520	0.715
0.55	0.539	0.513	0.571	0.531	0.710
0.6	0.547	0.518	0.573	0.541	0.707

Here, we focus on the new aspect, namely the similarities between the conditional performance score for normally and binary distributed endpoints. For normally distributed endpoints, Herrmann et al. [11] applied a *t-test* statistic with an asymptotic distribution $N(\Delta \sqrt{n/2},1)$. In this paper, we applied a normal approximation test with asymptotic distribution $N(\lambda \sqrt{n/2},1-1/4 \cdot \lambda ^{2})$. If we set $\Delta =\lambda$, the asymptotic test statistic distributions only differ by $1/4 \cdot \lambda ^{2}$ in the variance component. Given the values for $\lambda$ examined in this study, the difference in variance is between 0.00 and 0.09. Therefore, the asymptotic distributions of the respective test statistics are almost the same. As the considered recalculation rules are based on the asymptotic distribution of the test statistics, the behavior of the designs is similar for both endpoint types. Accordingly, the score results are also similar.

### Results APSAC simulation

**Fig. 3:**
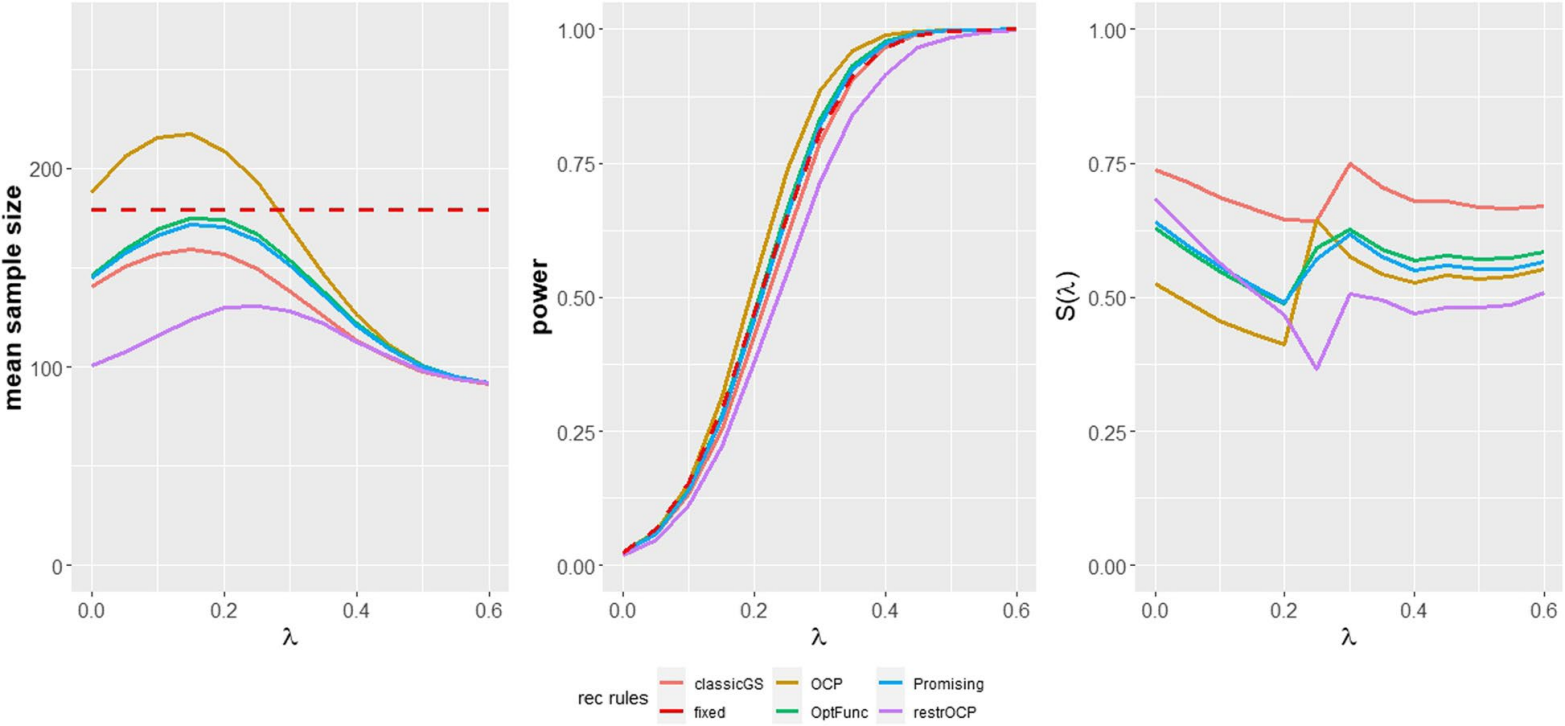
Performance measures for the recalculation rules in the APSAC simulation

The results of the APSAC simulation are provided in Table 4 and Fig. 3. It is remarkable, that the optimization function approach and the promising zone approach both achieve a higher power and a lower mean sample size than the fixed design in the area around the assumed standardized treatment effect of $\lambda =0.295$. This provides some strong arguments for the benefits of adaptive designs with sample size recalculation for this practical application. However, the global measures power and mean sample size do not fully reflect the usefulness of a design. By considering variation in the sample size and the observed conditional power, the conditional performance score leads to a different evaluation of the recalculation rules. Here, just like in the main simulation, the group sequential design performs best. This result can be attributed to less variation in the sample size, compared to the other designs with sample size recalculation.

**Table 4:** Score results $S(\lambda )$ for the recalculation rules in the APSAC simulation. The values in the table correspond to Fig. 3. The first stage sample size is set to $n_{1}=90$, the maximum sample size is set to $n_{max}=270$ with global one-sided significance level $\alpha = 0.025$, efficacy stopping boundaries were chosen according to Pocock [15] and binding futility stopping bound represented by $\alpha _{0}=0.5$. OCP: Observed conditional power approach; restrOCP: Restricted observed conditional power approach; Promising: Promising zone approach; OptFunc: Optimization function approach; classicGS: group sequential approach

$\varvec{\lambda }$	OCP	restrOCP	Promising	OptFunc	classicGS
0	0.524	0.684	0.640	0.630	0.737
0.05	0.491	0.624	0.598	0.589	0.714
0.1	0.457	0.566	0.557	0.549	0.687
0.15	0.432	0.514	0.523	0.517	0.666
0.2	0.411	0.467	0.491	0.488	0.645
0.25	0.646	0.366	0.572	0.593	0.644
0.3	0.577	0.508	0.617	0.628	0.749
0.35	0.544	0.495	0.576	0.591	0.706
0.4	0.527	0.470	0.552	0.570	0.681
0.45	0.542	0.482	0.559	0.579	0.680
0.5	0.536	0.480	0.552	0.572	0.669
0.55	0.539	0.487	0.553	0.575	0.667
0.6	0.553	0.509	0.568	0.586	0.671

The OCP and the restricted OCP perform worst according to the conditional performance score. Also with regard to the global performance measures, these recalculation rules appear problematic. The OCP requires significantly higher mean sample sizes than the other recalculation rules. The restricted OCP, on the other hand, requires the smallest mean sample size but leads to severe underpowering in the area around the assumed standardized treatment effect.

## Discussion

We found that the performance of recalculation rules for binary endpoints can be calculated in the same way as for normally distributed endpoints. We can define all components of the score (conditional power, recalculated sample size, fixed design sample size) for binary endpoints and we can calculate their location and variation parameters, which are required for the score definition by Herrmann et al. [11], who considered the case of normally distributed endpoints.

A difference between the case of normally distributed and binary endpoints lies in the parameter value used to represent the endpoint’s distribution. In the case of normally distributed endpoints, it is possible to represent the endpoint distribution by the standardized treatment effect $\Delta {:=}(\mu _{I}-\mu _{C})/(\sigma )$, where $\mu _{I}, \mu _{C}$ are the expected values in the intervention and control group and $\sigma$ is the standard deviation in both groups. For binary endpoints, we found that the endpoint distribution can be parameterized by the single parameter $\lambda {:=}(p_{I}-p_{C})/(\sqrt{\frac{p_{I}+p_{C}}{2}(1-\frac{p_{I}+p_{C}}{2})})$, which corresponds to the standardized treatment effect for binary endpoints. For all the considered recalculation rules, the results regarding the conditional performance score proved similar behavior for binary and normally distributed endpoints with the same standardized treatment effect ($\Delta =\lambda$).

The conditional performance score can now also be applied to trial scenarios with binary endpoints. The applying scientists need to specify ranges of plausible values for $p_{I}$ and $p_{C}$ and compare the score values of the considered adaptive designs over these plausible endpoint distributions. The parameterization in $\lambda$ simplifies this step, as ranges for $p_{I}$ and $p_{C}$ are combined into a single plausible interval in $\lambda$. Following our simulation approach, the scientists can then calculate the score values for each $\lambda$. If a recalculation rule proves to be superior over the whole interval in dependence of $\lambda$, this provides evidence for the suitability of the rule.

We derived the standardized treatment effect parameter $\lambda$ to allow for a one-dimensional parameterization of the normal approximation test statistic distribution and performance measures of the considered designs. For the normal approximation test, neither the expected value nor the variance of the asymptotic test statistic distribution is constant with regard to $p_I$ and $p_C$ (see Eq. (3)). As a consequence, this would require representing all performance values in dependence of two parameters (expected value and variance) if we had not found the one-dimensional parameterization by $\lambda$. An interesting question is what would change if we did not apply the normal approximation test but another test statistic. It is possible to apply a test statistic with an asymptotic normal distribution and a variance, which is constantly 1, for any $p_I$, $p_C$. This would, for example, be the case when applying a Wald test [22]. In this case, the asymptotic test statistic distribution has already a one-dimensional representation (with the expected value of the asymptotic normal distribution as the only distribution parameter) and the transformations we did in Appendix B to derive $\lambda$, would not be necessary. This presents another statistical testing strategy that could be applied in such application scenarios. However, it also requires the corresponding mathematical derivations for (conditional) power, fixed design’s sample size, conditional performance score etc. (i.e. all formulas depending on $\lambda$).

Regarding the definition of the score, we note that the four components (location and variation component of conditional power and sample size) could be weighted differently [23]. For our simulation, we choose an equal weighting. Scientists seeing higher relevance in specific components could change the weighting scheme in a suitable manner.

Furthermore, we presented a real-world trial example. We note that for the application it is beneficial to complement the conditional performance score with global performance measures. In the empirical analysis, we have seen that none of the considered recalculation rules behaves really favorable if the true effect $\lambda$ is much smaller than the assumed effect size (0.3 for the main simulation and 0.295 for the APSAC simulation), which was applied for the sample size calculation of the fixed design. Some of the designs would expose on average more than 100 patients per group to the trial, although there is little chance of success (global power far below 50%). Even though, for most of the considered adaptive designs, the average number of patients in such futile scenarios is lower than for the fixed design, this still does not represent a favorable result. This is a valuable insight from the global perspective. Even though sample size recalculation offers a variety of possibilities of addressing insecurities about the underlying effect size, large deviations can still have a significant impact on their respective power values.

Regarding further research, an extension of the conditional performance score to other endpoint types would likely also be possible, as the underlying criteria (sample size and conditional power) are not restricted to specific endpoint types. Having examined the case of normally distributed endpoints and binary endpoints, an obvious question is the extension to time-to-event endpoints. For such endpoints, the adaption of recalculation rules is likely to be challenging as sample size considerations for time-to-event are generally complicated.

In addition, it would also be possible to extend the research to the application of further recalculation rules. The recalculation rules considered in this paper are a collection of methods which have been suggested in previous literature. It would be possible to adapt these rules (e.g. by changing the parameter estimation approach at interim) and to examine additional rules. Also, other parameter choices in the applied recalculation rules would be possible (e.g. different values for $\gamma$ and $\beta _{0}^{prom}$). The score could theoretically be used for any recalculation rule, where we can calculate sample size and conditional power. The search for optimal recalculation rules is still ongoing.

### Supplementary Information

**Additional file 1.** Appendices.

## Data Availability

No original trial data are used in this work. Simulated data and software source code that support the findings of the simulation study can be found in the github repository https://github.com/bokelmab/cond_score_binary_endpoint.
