# Supplementary material for: Extension of a conditional performance score for sample size recalculation rules to the setting of binary endpoints
Source: BMC Med Res Methodol. 2024 Jan 19;24:15. doi: 10.1186/s12874-024-02150-4 (PMC10797857; doi:10.1186/s12874-024-02150-4)
Supplement: Supplementary file 1 — Additional file 1. Appendices. [file 12874_2024_2150_MOESM1_ESM.pdf]

## Appendix A: Distribution of the normal approximation test

In the following, we derive the distribution of the normal approximation test, given in Equation (3). Here, a right arrow with $d$ or $p$ on top denotes convergence in distribution and probability respectively.

For the numerator of the test statistic, it follows from the central limit theorem that

$$\sqrt{n}(\bar{X^I} - \bar{X^C}) \xrightarrow{\text{d}} N((p_I - p_C)\sqrt{n}, p_I(1-p_I) + p_C(1-p_C)).$$

For the denominator, it follows from the law of large numbers

$$\sqrt{\frac{\bar{X^I} + \bar{X^C}}{2}\left(1 - \frac{\bar{X^I} + \bar{X^C}}{2}\right)} \xrightarrow{\text{p}} \sqrt{\frac{p_I + p_C}{2}\left(1 - \frac{p_I + p_C}{2}\right)}.$$

Since the denominator converges against a constant, we can derive the asymptotic distribution of the normal approximation test statistic using slutsky's theorem

$$\sqrt{\frac{n}{2}} \frac{\bar{X^I} - \bar{X^C}}{\sqrt{\frac{\bar{X^I}+\bar{X^C}}{2}\left(1 - \frac{\bar{X^I}+\bar{X^C}}{2}\right)}} \xrightarrow{\text{d}} N\left(\frac{p_I - p_C}{\sqrt{(\frac{p_I+p_C}{2})(1 - \frac{p_I+p_C}{2})}}\sqrt{\frac{n}{2}}, \frac{p_I(1-p_I) + p_C(1-p_C)}{2(\frac{p_I+p_C}{2})(1 - \frac{p_I+p_C}{2})}\right).$$

## Appendix B: One-dimensional parameterization of the test statistic for binary endpoints

It is noteworthy that both the mean and the variance of $Z$ depend on the endpoint distribution parameters $p_I, p_C$ (see equation (3)). This property differs from the case of normally distributed endpoints analyzed by Herrmann et al.[1] where the variance of $Z$ is assumed to be constantly 1. However, with some transformation steps there exists a parameterization of the distribution from $Z$ by a single parameter. In the

following, we present the derivation using

$$\sigma_A^2 := p_I(1-p_I) + p_C(1-p_C) \text{ and}$$
$$\sigma_B^2 := (\frac{p_I+p_C}{2})(1-\frac{p_I+p_C}{2}),$$

which leads to the simplified representation

$$Z \sim N\left(\frac{p_I - p_C}{\sigma_B}\sqrt{\frac{n}{2}}, \frac{\sigma_A^2}{2\sigma_B^2}\right). \tag{B1}$$

Our goal is to find a parameter $\lambda$, which parameterizes the mean and variance component of the test statistic's distribution simultaneously. The parameter $\lambda := (p_I - p_C)/\sigma_B$ is a plausible candidate for such a parameterization. Hence, in the following, we demonstrate how the variance component $\sigma_A^2/(2\sigma_B^2)$ can be parameterized by $\lambda$.

It holds that

$$\begin{aligned}
2\sigma_B^2 - \sigma_A^2 &= (p_I+p_C)(1-\frac{p_I+p_C}{2}) - p_I(1-p_I) - p_C(1-p_C) \\
&= p_I + p_C - \frac{(p_I+p_C)^2}{2} - p_I + p_I^2 - p_C + p_C^2 \\
&= -\frac{(p_I+p_C)^2}{2} + p_I^2 + p_C^2 \\
&= -\frac{p_I^2 + 2p_Ip_C + p_C^2}{2} + p_I^2 + p_C^2 \\
&= \frac{p_I^2}{2} + \frac{p_C^2}{2} - p_Ip_C \\
&= \frac{(p_I-p_C)^2}{2}.
\end{aligned}$$

Hence,

$$\sigma_A^2 = 2\sigma_B^2 - \frac{(p_I-p_C)^2}{2}, \tag{B2}$$

and therefore

$$\begin{aligned}\frac{\sigma_A^2}{2\sigma_B^2} &= 1-\frac{1}{4}\frac{(p_I-p_C)^2}{\sigma_B^2}\\ &= 1-\frac{1}{4}\lambda^2.\end{aligned}$$

Plugging this result together with the definition of
$\lambda := (p_I - p_C)/(\sqrt{(\frac{p_I+p_C}{2})(1-\frac{p_I+p_C}{2})})$ into Equation (B1) leads to

$$Z \sim N\left(\lambda\sqrt{\frac{n}{2}}, 1-\frac{1}{4}\lambda^2\right). \tag{B3}$$

Our goal was to derive a representation of the asymptotic distribution of the normal approximation test, based on a single parameter. Note that Wassmer and Brannath[2] provide a related idea. They derive a single distribution parameter $\zeta$, which parameterizes the power of the normal approximation test. The definition of $\zeta$ by Wassmer and Brannath[2] differs from the definition of the parameter $\lambda$ in this paper. However, both are strongly related as they can represent concepts derived from the asymptotic test statistic distribution (like the power) by a single distribution parameter.

## Appendix C: Adaptive designs for binary endpoints

### C.1 Derivation of the conditional power

We set $\bar{w_1} := \frac{w_1}{\sqrt{w_1^2+w_2^2}}$ and $\bar{w_2} := \frac{w_2}{\sqrt{w_1^2+w_2^2}}$. Since the combined test statistic is given by

$$Z_{1+2} := \bar{w_1}Z_1 + \bar{w_2}Z_2, \tag{C4}$$

the conditional power corresponds to

$$\begin{aligned} CP_{\lambda}(z_1, n) &= P[Z_{1+2} > q_{1-\alpha_{1+2}}] \\ &= P[\bar{w_1} Z_1 + \bar{w_2} Z_2 > q_{1-\alpha_{1+2}}] \\ &= P[\bar{w_2} Z_2 > q_{1-\alpha_{1+2}} - \bar{w_1} Z_1] \\ &= P\left[Z_2 > \frac{q_{1-\alpha_{1+2}}}{\bar{w_2}} - \frac{\bar{w_1}}{\bar{w_2}} Z_1\right] \\ &= P\left[\frac{Z_2 - \lambda\sqrt{\frac{n}{2}}}{\sqrt{1-\frac{1}{4}\lambda^2}} > \frac{\frac{q_{1-\alpha_{1+2}}}{\bar{w_2}} - \frac{\bar{w_1}}{\bar{w_2}} Z_1 - \lambda\sqrt{\frac{n}{2}}}{\sqrt{1-\frac{1}{4}\lambda^2}}\right]. \end{aligned}$$

From Equation (4) it follows that

$$\frac{Z_2 - \lambda\sqrt{\frac{n}{2}}}{\sqrt{1-\frac{1}{4}\lambda^2}} \sim N(0,1). \tag{C5}$$

Hence, it holds that

$$CP_{\lambda}(z_1, n) = 1 - \Phi\left(\frac{\frac{q_{1-\alpha_{1+2}}}{\bar{w_2}} - \frac{\bar{w_1}}{\bar{w_2}} Z_1 - \lambda\sqrt{\frac{n}{2}}}{\sqrt{1-\frac{1}{4}\lambda^2}}\right) \tag{C6}$$

$$= 1 - \Phi\left(\frac{\frac{\sqrt{w_1^2+w_2^2}}{w_2} q_{1-\alpha_{1+2}} - \frac{w_1}{w_2} Z_1 - \lambda\sqrt{\frac{n}{2}}}{\sqrt{1-\frac{1}{4}\lambda^2}}\right). \tag{C7}$$

## C.2 Derivation of the recalculated sample size formulas

The basic idea of the recalculation rules considered in the simulation is to calculate the sample size $\tilde{n}$, such that the observed conditional power reaches a predefined targeted value $CP_{\hat{\lambda}}(z_1, \tilde{n}) = 1 - \beta$. Note that the formula for the observed conditional power corresponds to the formula for the true conditional power, with the true parameter $\lambda$ replaced by the estimated parameter $\hat{\lambda}$.[3] This leads to

$$\Phi\left(\frac{\frac{\sqrt{w_1^2+w_2^2}}{w_2} q_{1-\alpha_{1+2}} - \frac{w_1}{w_2} Z_1 - \hat{\lambda}\sqrt{\frac{\tilde{n}}{2}}}{\sqrt{1-\frac{1}{4}\hat{\lambda}^2}}\right) = \beta$$

$$
\begin{aligned}
\frac{\frac{\sqrt{w_1^2+w_2^2}}{w_2}q_{1-\alpha_{1+2}} - \frac{w_1}{w_2}Z_1 - \hat{\lambda}\sqrt{\frac{\tilde{n}}{2}}}{\sqrt{1-\frac{1}{4}\hat{\lambda}^2}} &= q_\beta \\
\frac{\sqrt{w_1^2+w_2^2}}{w_2}q_{1-\alpha_{1+2}} - \frac{w_1}{w_2}Z_1 - \hat{\lambda}\sqrt{\frac{\tilde{n}}{2}} &= q_\beta\sqrt{1-\frac{1}{4}\hat{\lambda}^2} \\
\frac{\sqrt{w_1^2+w_2^2}}{w_2}q_{1-\alpha_{1+2}} - \frac{w_1}{w_2}Z_1 - q_\beta\sqrt{1-\frac{1}{4}\hat{\lambda}^2} &= \hat{\lambda}\sqrt{\frac{\tilde{n}}{2}} \\
\frac{\frac{\sqrt{w_1^2+w_2^2}}{w_2}q_{1-\alpha_{1+2}} - \frac{w_1}{w_2}Z_1 - q_\beta\sqrt{1-\frac{1}{4}\hat{\lambda}^2}}{\hat{\lambda}}\sqrt{2} &= \sqrt{\tilde{n}} \\
\left(\frac{\frac{\sqrt{w_1^2+w_2^2}}{w_2}q_{1-\alpha_{1+2}} - \frac{w_1}{w_2}Z_1 - q_\beta\sqrt{1-\frac{1}{4}\hat{\lambda}^2}}{\hat{\lambda}}\right)^2 2 &= \tilde{n}.
\end{aligned}
$$

With $\hat{\lambda} = \sqrt{\frac{2}{n_1}}Z_1$ this leads to

$$
n_1\left(\frac{\frac{\sqrt{w_1^2+w_2^2}}{w_2}q_{1-\alpha_{1+2}} - \frac{w_1}{w_2}Z_1 - q_\beta\sqrt{1-\frac{1}{2n_1}Z_1^2}}{Z_1}\right)^2 = \tilde{n} \qquad \text{(C8)}
$$

$$
n_1\left(\frac{\frac{\sqrt{w_1^2+w_2^2}}{w_2}q_{1-\alpha_{1+2}} - q_\beta\sqrt{1-\frac{1}{2n_1}Z_1^2}}{Z_1} - \frac{w_1}{w_2}\right)^2 = \tilde{n} \qquad \text{(C9)}
$$

Note that in the above equation, $\tilde{n}$ is not necessarily an integer. Therefore, the result needs to be rounded up to get the recalculated sample size.

From Formula (C9) for $\tilde{n}$ and Formula (C7) for $CP_{\hat{\lambda}}(z_1, n)$, the formulas of all the considered recalculation rules can be derived. To simplify the formulas we define $N_{rec}^{max} := n_{max} - n_1$. Below, we provide the formulas for the observed conditional power approach, the restricted observed conditional power approach, and the promising zone approach. Remember that $N_{rec}$ refers to the second stage sample

size per group, such that the total sample size per group would be $n_1 + N_{rec}$.

$$N_{rec}^{OCP}(z_1) = \begin{cases} \tilde{n} \text{ if } \tilde{n} \leq N_{rec}^{max} \\ N_{rec}^{max} \text{ else} \end{cases}$$

$$N_{rec}^{restrOCP}(z_1) = \begin{cases} 0 \text{ if } CP_{\hat{\lambda}}(z_1, N_{rec}^{max}) < 1 - \beta_0^{restrOCP} \\ \tilde{n} \text{ if } \tilde{n} \leq N_{rec}^{max} \\ N_{rec}^{max} \text{ if } CP_{\hat{\lambda}}(z_1, N_{rec}^{max}) \geq 1 - \beta_0^{restrOCP} \text{ and } \tilde{n} > N_{rec}^{max} \end{cases}$$

$$N_{rec}^{Prom}(z_1) = \begin{cases} n_{ini}^{Prom} \text{ if } CP_{\hat{\lambda}}(z_1, n_{ini}^{Prom}) < 1 - \beta_0^{Prom} \\ \tilde{n} \text{ if } 1-\beta > CP_{\hat{\lambda}}(z_1, n_{ini}^{Prom}) \geq 1 - \beta_0^{Prom} \text{ and } \tilde{n} \leq N_{rec}^{max} \\ N_{rec}^{max} \text{ if } 1-\beta > CP_{\hat{\lambda}}(z_1, n_{ini}^{Prom}) \geq 1 - \beta_0^{Prom} \text{ and } \tilde{n} > N_{rec}^{max} \\ n_{ini}^{Prom} \text{ if } CP_{\hat{\lambda}}(z_1, n_{ini}^{Prom}) \geq 1 - \beta \end{cases}$$

The recalculated sample size for the optimization function approach is defined via the formulas

$$N_{rec}^{OptFunc}(z_1) = argmax_{n \in [n_{ini}^{OptFunc}, N_{rec}^{max}]} f_\gamma(z_1, n)$$
$$f_\gamma(z_1, n) := CP_{\hat{\lambda}}(z_1, n) - \gamma(n - n_{ini}^{OptFunc}).$$

The formulas contain, in addition to the type 2 error rate $\beta$, some more parameters $\beta_0^{resrOCP}, n_{ini}^{Prom}, n_{ini}^{OptFunc}, \beta_0^{Prom}, \gamma$, which need to be specified when using the approaches. Our choices for these parameters are given in Section 4.

C.3 Derivation of a formula for the power calculation

In the simulations of the main manuscript, we needed to calculate the global power of the considered designs. To calculate the global power, we used the decomposition

$$\begin{aligned} Pow &= P[Z_1 \geq q_{1-\alpha_1}] + P[q_{1-\alpha_0} \leq Z_1 < q_{1-\alpha_1} \wedge Z_{1+2} \geq q_{1-\alpha_{1+2}}] \\ &= E[\mathbb{1}_{Z_1 \geq q_{1-\alpha_1}}] + E[\mathbb{1}_{q_{1-\alpha_0} \leq Z_1 < q_{1-\alpha_1}} \cdot \mathbb{1}_{Z_{1+2} \geq q_{1-\alpha_{1+2}}}] \\ &= E[\mathbb{1}_{Z_1 \geq q_{1-\alpha_1}}] + E[E[\mathbb{1}_{q_{1-\alpha_0} \leq Z_1 < q_{1-\alpha_1}} \cdot \mathbb{1}_{Z_{1+2} \geq q_{1-\alpha_{1+2}}} | Z_1]] \\ &= E[\mathbb{1}_{Z_1 \geq q_{1-\alpha_1}}] + E[\mathbb{1}_{q_{1-\alpha_0} \leq Z_1 < q_{1-\alpha_1}} \cdot E[\mathbb{1}_{Z_{1+2} \geq q_{1-\alpha_{1+2}}} | Z_1]] \\ &= E[\mathbb{1}_{Z_1 \geq q_{1-\alpha_1}}] + E[\mathbb{1}_{q_{1-\alpha_0} \leq Z_1 < q_{1-\alpha_1}} \cdot CP_\lambda(Z_1, N_{rec})]. \end{aligned}$$

Hence, the global power can be estimated, via simulation, according to

$$Pow(\lambda) = \frac{1}{N_{sim}} \sum_{i=1}^{N_{sim}} (\mathbb{1}_{Z_{1,i} \geq q_{1-\alpha_1}} + \mathbb{1}_{q_{1-\alpha_0} \leq Z_{1,i} < q_{1-\alpha_1}} \cdot CP_\lambda(Z_{1,i}, N_{rec,i})),$$

where $Z_{1,i}$ denote the values of the interim test statistic of simulation $i \in \{1, 2, ..., N_{sim}\}$.

## Appendix D: Fixed design sample size $n_{fix}$

Following Kieser[4], the fixed design sample size per group (given equal group sizes) for a normal approximation test on binary endpoints is given by

$$n_{fix} = \frac{(q_{1-\alpha_{1+2}} \sqrt{2\sigma_B^2} + q_{1-\beta} \sqrt{\sigma_A^2})^2}{(p_I - p_C)^2}.$$

We know from Equation (B2) that

$$\sigma_A^2 = 2\sigma_B^2 - \frac{(p_I - p_C)^2}{2}.$$

Plugging this in to the above equation leads to

$$
\begin{aligned}
n_{fix} &= \frac{(q_{1-\alpha_{1+2}}\sqrt{2\sigma_B^2} + q_{1-\beta}\sqrt{2\sigma_B^2 - \frac{(p_I-p_C)^2}{2}})^2}{(p_I - p_C)^2} \\
&= \left(q_{1-\alpha_{1+2}}\sqrt{2\frac{\sigma_B^2}{(p_I-p_C)^2}} + q_{1-\beta}\sqrt{2\frac{\sigma_B^2}{(p_I-p_C)^2} - \frac{1}{2}}\right)^2 .
\end{aligned}
$$

We defined $\lambda := \frac{p_I - p_C}{\sigma_B}$, which leads to

$$
n_{fix} = \left(\sqrt{2}q_{1-\alpha_{1+2}}\frac{1}{\lambda} + q_{1-\beta}\sqrt{\frac{2}{\lambda^2} - \frac{1}{2}}\right)^2 .
$$

Note that the equation does not necessarily yield whole numbers. Hence, the recalculated sample size needs to be obtained by rounding up to the next whole number.

## Appendix E: Deviations from asymptotic properties

In the previous sections we assumed a large enough sample size for an approximate normal distribution of the considered test statistic $Z$. This assumption was crucial for the derivation of the futility and efficacy boundaries, so accordingly, the type I error rate of the considered designs depends on this assumption. Moreover, the one-dimensional parameterization of the power, mean sample size, and the conditional performance score was derived under this normality assumption. Since we know that in practice, due to a limited sample size, the normality assumption holds at most approximately, it is necessary to check whether the derived theory is applicable.

### E.1 type I error rate

Asymptotically, the type I error rate should be 2.5%. However, this only holds if $Z_1$ and $Z_2$ follow exactly the derived asymptotic normal distribution. In practice, $Z_1$ and $Z_2$ only approximate the asymptotic distribution. How well they approximate the asymptotic distribution depends on the sample sizes $n_1, N_{rec}$ and the proportion of events $p_C$. (Note that under the null hypothesis, it holds $p_I = p_C$, which is why $p_C$

is sufficient to characterize the endpoint's distribution.) Since $N_{rec}$ is stochastic in our setting, we only need to consider the type I error rate for various combinations of $n_1$ and $p_C$. The results are given in Figure S1. The results were generated by the simulation described in Section 4.2. The type I error rate corresponds to a simulation of the true global power for a standardized treatment effect of $\lambda = 0$.

We observe that for small values of $p_C$, our designs tend to have an error rate below 2.5%. Only for large values of $p_C$ exceeding of the type I error rate occurs. For large values of $n_1$, the designs barely exceed the specified type I error rate, which is why we can conclude that the type I error rate does not pose a problem in our considered main setting with $n_1 = 50$ and $p_C = 0.3$. Note, that the relatively small type I error rate for the restricted OCP is due to the additional option to stop for futility if $CP_{\hat{\lambda}}(Z_1, n_{max})$ is below the specified threshold.

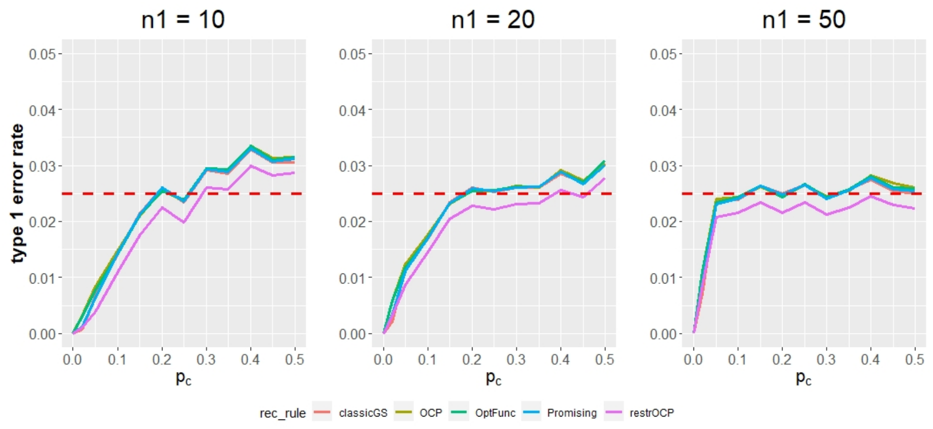


**Figure S1** type I error rate for different values of $n_1$ and $p_c$.

## E.2 Power, mean sample size, and conditional performance score

In the following, we want to check whether, in the finite sample case, the single parameter $\lambda$ is sufficient to parameterize the (global) power, mean sample size, and conditional performance score of the considered recalculation rules. Therefore, we check whether the performance measures, visualized as a function of $\lambda$, behave differently for different values of $p_C$. If so, it is not sufficient to present results

only depending on $\lambda$ and a differentiated representation for different values of $p_C$ is necessary. To limit the scope of the analysis, we keep $n_1 = 50$ fixed.

We observe in Figures S2, S3, S4, S5 and S6 that differences for different values of $p_C$ are rather marginal. Accordingly, the relationship between $\lambda$ and the mean sample size, the power as well as the conditional performance score does almost not depend on $p_C$. Accordingly, the 1-dimensional parameterization of mean sample size, power, and conditional performance score by $\lambda$ is approximately correct for the considered setting.

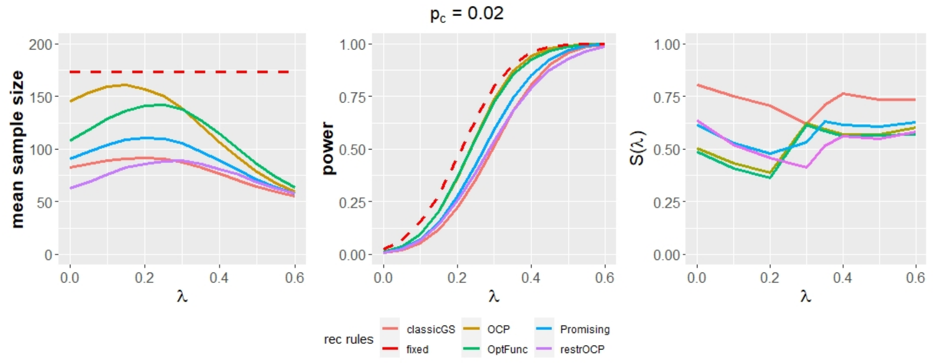


**Figure S2** Performance measures for $p_c = 0.02$ and different values of $\lambda$

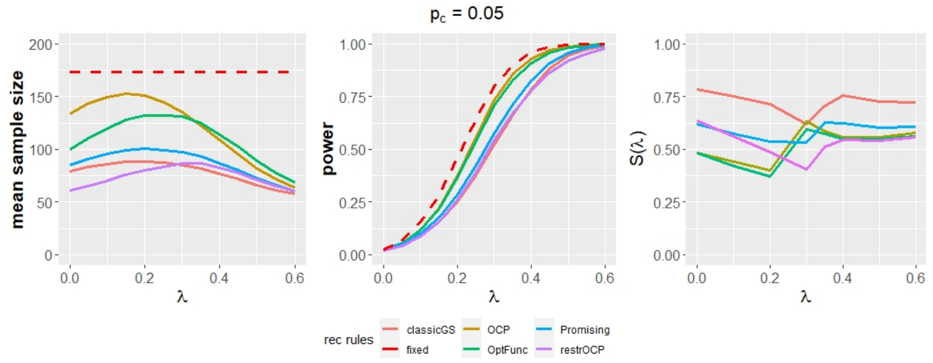


**Figure S3** Performance measures for $p_c = 0.05$ and different values of $\lambda$

**Author details**

**References**


1. Herrmann, C., Pilz, M., Kieser, M., Rauch, G.: A new conditional performance score for the evaluation of adaptive group sequential designs with sample size recalculation. Statistics in Medicine **39**(15), 2067–2100

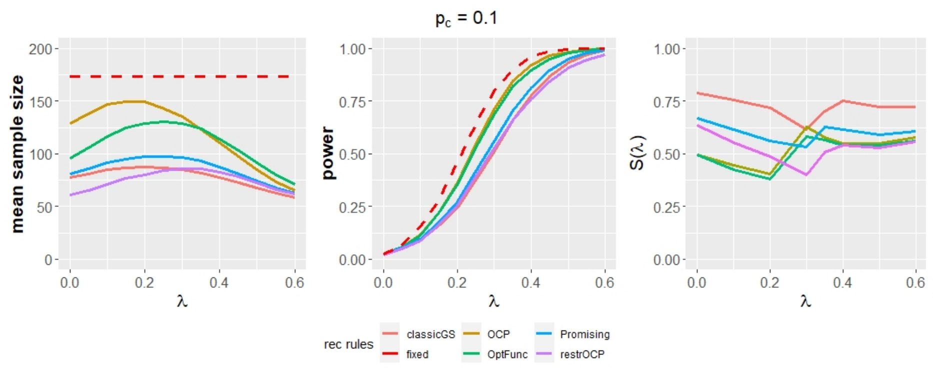


**Figure S4** Performance measures for $p_c = 0.1$ and different values of $\lambda$

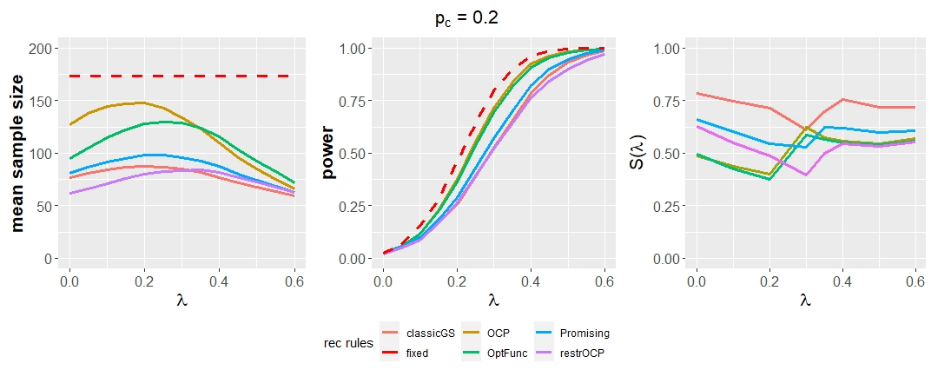


**Figure S5** Performance measures for $p_c = 0.2$ and different values of $\lambda$

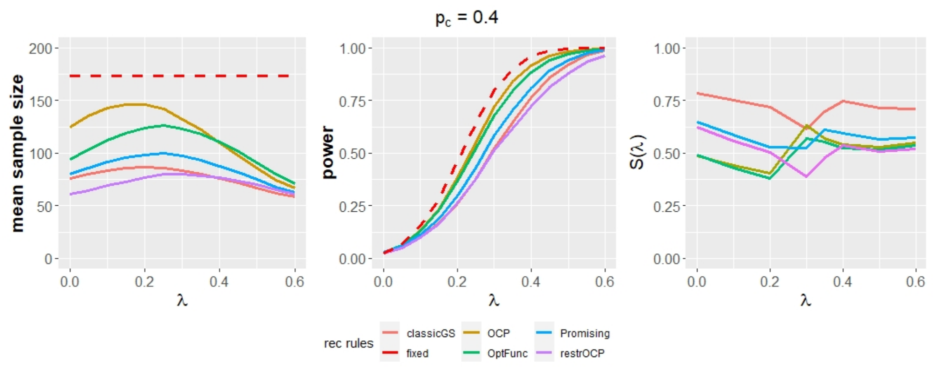


**Figure S6** Performance measures for $p_c = 0.4$ and different values of $\lambda$


(2020)

2. Wassmer, G., Brannath, W.: Group sequential and confirmatory adaptive designs in clinical trials., pp. 110–120. Springer (2016). Chap. Binary Response
3. Wassmer, G., Brannath, W.: Group sequential and confirmatory adaptive designs in clinical trials., pp. 178–184.

Springer (2016). Chap. On the Parameter Value Used in the Conditional Power and Sample Size Calculation

4. Kieser, M.: Methods and applications of sample size calculation and recalculation in clinical trials, pp. 48–51. Springer (2020). Chap. Comparison of Two Groups for Binary Outcomes and Test for Difference or Superiority
